# Prognostic Significance of Blood Transfusion in Elderly Patients with Primary Diffuse Large B-Cell Lymphoma

**DOI:** 10.1155/2018/6742646

**Published:** 2018-03-20

**Authors:** Liping Fan, Danhui Fu, Jinquan Hong, Haobo Huang, Wenqian He, Feng Zeng, Qiuyan Lin, Qianling Xie

**Affiliations:** ^1^Department of Blood Transfusion, Fujian Medical University Union Hospital, Gulou District, Fuzhou City, Fujian Province 350001, China; ^2^Department of Hematology, Fujian Medical University Union Hospital and Fujian Institute of Hematology, Gulou District, Fuzhou City, Fujian Province 350001, China; ^3^Department of Anesthesia, The Second Affiliated Hospital of Fujian Medical University, Licheng District, Quanzhou City, Fujian Province 362000, China; ^4^Department of Anesthesia, The Macare Women's Hospital of Quanzhou Municipal, Quanzhou City, Fujian Province 362000, China

## Abstract

The current study sought to evaluate whether blood transfusions affect survival of elderly patients with primary diffuse large B-cell lymphoma (DLBCL). A total of 104 patients aged 60 years and over were enrolled and divided into two groups: 24 patients who received transfusions and 80 patients who did not. Statistical analyses showed significant differences in LDH levels, platelet (Plt) counts, and hemoglobin (Hb) and albumin (Alb) levels between the two groups. Univariate analyses showed that LDH level ≥ 245 IU/L, cell of origin (germinal center/nongerminal center), and blood transfusion were associated with both overall survival (OS) and progression-free survival (PFS). Higher IPI (3–5), Alb level < 35 g/L, and rituximab usage were associated with OS. Appearance of B symptoms was associated with PFS. Multivariate analyses showed that cell of origin and rituximab usage were independent factors for OS and LDH level was an independent factor for PFS. Blood transfusion was an independent factor for PFS, but not for OS. Our preliminary results suggested that elderly patients with primary DLBCL may benefit from a restrictive blood transfusion strategy.

## 1. Introduction

Diffuse large B-cell lymphoma (DLBCL) is the most common subtype of non-Hodgkin's lymphoma. Chemotherapy combined with immunotherapy was the most important therapy for DLBCL. Age > 60 years is a poor prognostic factor in lymphoma, as age associated comorbidity and suboptimal performance status lead to intolerance of chemoimmunotherapy [1]. Hematopoietic suppression induced by chemoimmunotherapy was a common adverse effect, leading to increased supportive methods for patients with age > 60 years compared to those with age < 60 years, including administration of blood components.

In recent years, several studies showed that transfusion of whole blood and blood components affected overall survival (OS) or progression-free survival (PFS) of patients with solid tumors [2–11]. However, few studies showed effects of blood transfusion on survival of patients with hematological malignancy [12–15]. To date, no study was found to elucidate the effect of blood transfusion on survival of elderly patients with primary DLBCL.

In this study, we reviewed the medical records and follow-up data of elderly patients with primary DLBCL in our hospital to elucidate the effects of blood transfusion on OS and PFS.

## 2. Materials and Methods

### 2.1. Ethics Statement

The ethics committee of Fujian Medical University Union Hospital approved this study. As this study was retrospective and did not affect patients' treatments, written informed consent from patients was not sought.

### 2.2. Study Design

Patients over 60 years old were defined as elderly. Chemotherapy patients received the R-CHOP regimen (rituximab, cyclophosphamide, doxorubicin, vincristine, and prednisone) or CHOP. Elderly patients with primary DLBCL who received more than 2 courses of chemotherapy and had complete follow-up data between June 2012 and May 2016 at our hospital were included in this study.

### 2.3. Acquisition and Definition of Data

In this study, data were collected from the medical records of elderly patients with primary DLBCL from 1 June 2012 to 31 May 2016 at Fujian Medical University Union Hospital, Fujian Province, China. Patients who received less than 2 courses of chemotherapy, those without complete data of clinicopathological characteristics, and those without complete follow-up data were excluded.

Diagnosis was made via tissue biopsy according to the World Health Organization (WHO) classification. Clinical event end points, such as disease progression and relapse, were evaluated by use of standards described previously [16]. OS was measured from the date on which the patient started treatment to the date of death or last follow-up. Death from all causes was included. PFS was measured from the date on which the patient started treatment to the date of disease progression, relapse, or death, whichever came first. Survival time was measured until 31 December 2016.

Patients that received more than 2 units of packed red blood cells (RBC), more than 1 unit (≥2 × 10^11^ platelets per unit) of apheresis platelets (Plt), or more than 15 ml/kg of fresh frozen plasma (FFP) during chemotherapy were categorized as blood transfusion group. All blood components were leukocyte reduced (leukocyte numbers < 5 × 10^5^ per bag). The storage duration of packed RBC units ranged from 5 to 34 days. The storage duration of apheresis Plt units and FFP was limited to 5 days and one year, respectively. The decisions to transfuse in elderly patients with primary DLBCL were made based on the treating doctors' judgment and guided by our hospital's technical manuals of clinical blood transfusion and hemotherapy decisions from the technical manual of American Association of Blood Banks (AABB) [17]. Technical manuals of clinical blood transfusion in our hospital have been previously described [13].

### 2.4. Statistical Analysis

All statistical analyses were performed using the software SPSS 19.0 for Windows. The chi-square test and independent *t*-test were used to analyze categorical and continuous variables of patients between the group receiving transfusions and the group that did not, respectively. The Kaplan-Meier method was used for calculating survival for PFS and OS, and the log-rank test was used for analyzing the differences among these survival curves. Multivariate analysis was performed using the Cox regression models to further evaluate all the significant prognostic factors found in the univariate analysis. Two-sided *P* values of <0.05 were considered statistically significant.

## 3. Results

### 3.1. Patient Data

The study included 104 elderly patients with primary DLBCL. During the follow-up period, 30 deaths occurred. The median follow-up interval of all patients was 14.1 (range from 2.0 to 55.8) months, comprising 18.6 (range from 6.8 to 55.8) months for survivors and 10.0 (range from 2.0 to 21.0) months for deceased. The characteristics of all patients before treatment are listed in Table 1.

Of the 104 inpatients, 24 patients (23.08%) received a blood transfusion and 80 patients (76.92%) did not. Erythropoiesis-stimulating agents (ESAs) were not used in all patients. In the transfused group, significantly more patients had higher LDH levels and lower platelet (Plt) counts and hemoglobin (Hb) and serum albumin (Alb) levels. There were no significant differences in age, gender, Ann Arbor staging, extranodular involvement, bone marrow involvement, B symptoms, IPI, cell of origin (GC/non-GC), and rituximab usage between the two groups.

### 3.2. Prognostic Factors of OS and PFS

**Figure 1:**
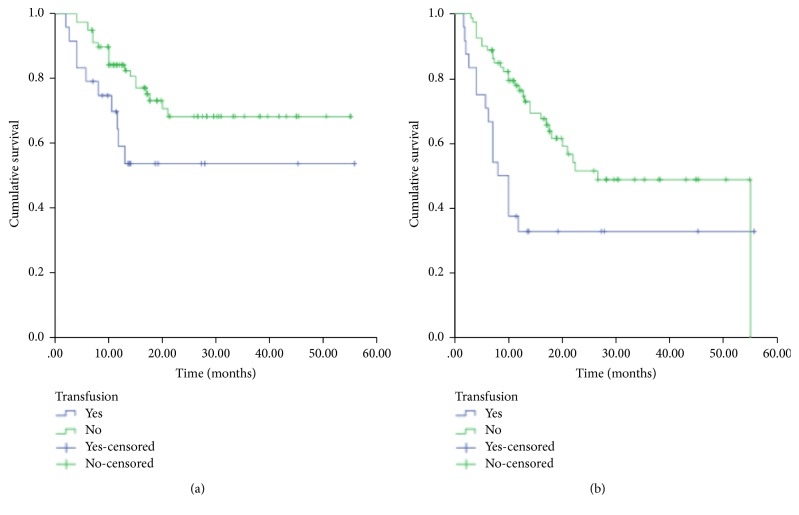
Kaplan-Meier curve of overall survival (a) and progression-free survival (b) of elderly patients with primary DLBCL who did or did not receive a blood transfusion.

Univariate analyses showed that patients with higher LDH levels (≥245 IU/L), higher IPI (3–5), GCB, lower serum Alb level (<35 g/L), or receiving blood transfusion had shorter OS than others in this cohort (Table 2, Figure 1(a)). Patients who received rituximab had longer OS (Table 2). Multivariate analyses showed that cell of origin (GC/non-GC) and rituximab usage were independent prognostic factors for OS in elderly patients with primary DLBCL, but blood transfusion was not an independent prognostic factor for OS in elderly patients with primary DLBCL (Table 3).

Regarding PFS, univariate analyses showed that patients with B symptoms, higher LDH levels (≥245 IU/L), or receiving blood transfusion had shorter PFS than others in this cohort (Table 2, Figure 1(b)). Patients with GCB had longer PFS (Table 2). Multivariate analyses showed that LDH levels and blood transfusion were independent prognostic factors for PFS in elderly patients with primary DLBCL (Table 3).

### 3.3. Discussion

DLBCL is a common lymphoma of hematological malignancy. Chemoimmunotherapy is the main therapy for DLBCL. Inhibition of hematopoietic function was a common side effect during chemotherapy. In order to increase patients' tolerance for chemoimmunotherapy, supportive care, including blood transfusion, becomes necessary [1].

Several studies have shown that during storage duration, blood cell products released factors and materials that may affect immune cells, such as monocytes, T lymphocytes, and natural killer cells, modulating recipients' immune systems [18–20]. These factors and materials also affect tumor cells [21]. So, theoretically, blood transfusion may affect the prognosis of patients with cancer. In fact, the effect of blood transfusion on the survival of patients with malignant diseases remains controversial. In the last decades, several studies showed that blood transfusion is an independent poor prognostic factor for survival in patients with many kinds of solid tumor [3–8, 10]. Others showed that blood transfusion did not affect survival in patients with gastric or bladder cancer [9, 22–24]. Heterogeneity of disease and therapy among patients can possibly explain these phenomena. However, nothing was found to elucidate the effect of blood transfusion on survival of elderly patients with primary DLBCL.

In the current study, we found significant differences in serum LDH levels, Alb levels, Plt counts, and Hb levels before treatment between the groups that did and did not receive transfusions. These may indicate the severity of disease and the requirement for blood components transfusion. In univariate analysis, we found that serum LDH ≥ 245 IU/L, higher IPI, B cells originated from GC, serum Alb < 35 g/L, utilization of rituximab, and blood transfusion were correlated with OS. With regard to PFS, B symptoms, serum LDH ≥ 245 IU/L, B cells originating from GC, and blood transfusion were correlated. We also found that Plt count and Hb level before treatment were not correlated with OS.

In multivariate analysis based on significant prognostic factors found in the univariate analysis, we found that B lymphoma cell originated from GC and utilization of rituximab were independent prognostic factors for OS. These results were in accordance with those previously described. IPI, which is commonly recognized as a prognostic predictor for patients with lymphoma, was not an independent prognostic factor for OS in our study cohort. As age > 60 years was reported as an important poor prognostic factor for lymphoma and was one of the main factors that constitutes IPI, IPI may have a reduced prognostic efficiency in our study cohort. We also found that blood transfusion did not affect OS. As far as PFS was concerned, serum LDH levels and blood transfusion were independent prognostic factors for PFS.

Selection of therapeutics is an important factor for surviving lymphoma. Chemoimmunotherapy, containing rituximab, cyclophosphamide, and doxorubicin, is the most prevalent and efficient regimen for DLBCL. It was reported that the cytotoxic effect of some drugs such as cyclophosphamide and doxorubicin can be influenced by hypoxia induced by severe anemia [25], so timely reversal of hypoxia may improve the effectiveness of chemoimmunotherapy. In the meantime, protein in plasma, such as Alb, acts as a drug carrier, and lower serum Alb levels may influence the metabolism and cytotoxicity of drugs in vivo [26, 27]. Therefore, blood transfusion is an important therapeutic method for patients undergoing chemotherapy, including patients with DLBCL. Otherwise, blood transfusion offers risks for recipients, such as hemolytic transfusion reactions, allergic transfusion reactions, transfusion-related acute lung injury, and transfusion-associated graft-versus-host disease. Therefore, the threshold for blood transfusion should be considered cautiously. Currently, the guidelines with regard to thresholds for blood components transfusion differ by country, as described previously [13].

In our study, though blood transfusion was associated with decreased OS and PFS in elderly patients with primary DLBCL, it was not an independent poor prognostic factor for OS but was an independent poor prognostic factor for PFS. Our study had two main limitations. First, our sample size of patients receiving transfusions was too small to generalize the results. Second, many of the patients receiving chemotherapy did not receive rituximab, a first-line component in many chemotherapy strategies. A prospective study with a larger sample size, including patients receiving transfusions and all patients undergoing chemotherapy compounded with rituximab, is needed to improve the results obtained from this study.

As blood transfusion was an independent poor prognostic factor for PFS and negatively associated with OS, a restricted transfusion strategy may be used for decisions about blood transfusion in elderly patients with primary DLBCL. In recent years, because some studies showed that administering ESAs could reduce the number of RBC transfusions, we recommended it as an adjunct to transfusion or an alternative in elderly patients with primary DLBCL. Recently, Greener et al. found that washed transfusion in patients with acute myeloid leukemia improved their clinical outcomes [28]. Therefore, we suggest that washed RBC may be a better option for those patients who need a blood transfusion immediately.

## Figures and Tables

**Table 1:** Characteristics of elderly DLBCL patients who did and did not receive blood transfusions.

Characteristic		Transfused	Nontransfused	*P*
		(*n* = 24) (%)	(*n* = 80) (%)	
Age, years	Mean	68.42 ± 0.90	68.74 ± 0.74	0.8246
Gender	Male	15 (62.5)	50 (62.5)	1.000
	Female	9 (37.5)	30 (37.5)	
Ann Arbor staging	I-II	7 (29.2)	29 (36.3)	0.6282
	III-IV	17 (70.8)	51 (63.7)	
Extranodular involvement	Yes	8 (33.3)	33 (41.3)	0.6348
	No	16 (66.7)	47 (58.7)	
Bone marrow involvement	Yes	1 (4.2)	2 (2.5)	0.5488
	No	23 (95.8)	78 (97.5)	
B symptoms	Yes	6 (25.0)	7 (8.8)	0.0708
	No	18 (75.0)	73 (91.2)	
LDH (IU/L)	≥245	15 (62.5)	27 (33.8)	0.0172
	<245	9 (37.5)	53 (66.2)	
IPI	0–2	8 (33.3)	37 (46.3)	0.3487
	3–5	16 (66.7)	43 (53.7)	
Cell of origin	GCB	5 (20.8)	32 (40.0)	0.0955
	Non-GCB	19 (79.2)	48 (60.0)	
Plt	≥100 × 10^9^/L	20 (83.3)	77 (96.2)	0.0479
	<100 × 10^9^/L	4 (16.7)	3 (3.8)	
Hb (g/L)	≥100	12 (50.0)	73 (91.2)	<0.0001
	<100	12 (50.0)	7 (8.8)	
Alb (g/L)	≥35	8 (33.3)	59 (73.8)	0.0005
	<35	16 (66.7)	21 (26.2)	
Rituximab	Yes	14 (58.3)	56 (70.0)	0.3255
	No	10 (41.7)	24 (30.0)	

LDH: lactate dehydrogenase; IPI: International Prognostic Index; Hb: hemoglobin; Alb: albumin.

**Table 2:** Univariate analysis of prognostic factors for survival time in elderly patients with primary DLBCL.

Characteristics		*n*	OS (mean)	Log-rank test	*P*	PFS (mean)	Log-rank test	*P*
Gender	Male	65	36.99	2.314	0.128	30.63	0.227	0.634
	Female	39	37.26			27.41		
Ann Arbor staging	I-II	36	45.83	3.078	0.079	34.71	0.675	0.411
	III-IV	68	36.47			29.14		
Extranodular involvement	Yes	41	38.71	0.555	0.456	30.26	0.274	0.601
	No	63	38.34			30.01		
Bone marrow involvement	Yes	3	29.83	1.028	0.311	36.30	0.425	0.514
	No	101	18.71			30.97		
B symptoms	Yes	13	23.37	3.105	0.078	15.95	5.052	0.025
	No	91	40.79			32.34		
LDH (IU/L)	≥245	42	33.63	3.907	0.048	25.46	6.044	0.014
	<245	62	43.42			34.89		
IPI	0–2	45	45.85	4.775	0.029	33.60	0.716	0.398
	3–5	59	35.43			29.46		
Cell of origin	GCB	37	39.92	7.312	0.007	31.21	4.139	0.042
	Non-GCB	67	34.22			27.77		
Plt	≥100 × 10^9^/L	97	39.72	0.008	0.931	30.92	0.057	0.812
	<100 × 10^9^/L	7	33.80			27.04		
Hb (g/L)	≥100	85	40.61	0.993	0.319	32.10	1.906	0.167
	<100	19	30.96			22.75		
Albumin (g/L)	≥35	67	43.85	5.822	0.016	33.32	3.498	0.061
	<35	37	32.61			26.81		
Rituximab	Yes	70	43.32	5.087	0.004	33.78	3.638	0.056
	No	34	30.43			23.82		
Blood transfusion	Yes	24	33.55	4.568	0.033	22.48	8.061	0.005
	No	80	41.53			33.65		

**Table 3:** Multivariate analysis of prognostic factors for survival time in elderly patients with primary DLBCL.

Covariates	Overall survival				Progression-free survival			
	Coefficient	SE	HR (95% CI)	*P*	Coefficient	SE	HR (95% CI)	*P*
B symptoms	N.A.	N.A.	N.A.	N.A.	−0.702	0.378	0.496 (0.236–1.040)	0.064
LDH	0.068	0.408	1.071 (0.482–2.380)	0.867	0.638	0.300	1.893 (1.052–3.405)	0.033
IPI	0.515	0.446	1.674 (0.698–4.014)	0.249	N.A.	N.A.	N.A.	N.A.
Cell of origin	1.348	0.494	3.848 (1.460–10.138)	0.006	0.428	0.337	1.534 (0.792–2.970)	0.204
Albumin	−0.568	0.376	0.567 (0.271–1.184)	0.131	N.A.	N.A.	N.A.	N.A.
Rituximab	0.922	0.367	2.515 (1.225–5.167)	0.012	N.A.	N.A.	N.A.	N.A.
Blood transfusion	−0.327	0.424	0.721 (0.314–1.654)	0.440	−0.654	0.318	0.520 (0.279–0.970)	0.040

LDH: lactate dehydrogenase; IPI: International Prognostic Index; Hb: hemoglobin; Alb: albumin; SE: standard error; HR: hazard ratio; CI: confidence interval; N.A.: not available.
